# The Calcineurin Inhibitor Tacrolimus Specifically Suppresses Human T Follicular Helper Cells

**DOI:** 10.3389/fimmu.2018.01184

**Published:** 2018-05-31

**Authors:** Elizabeth F. Wallin, Danika L. Hill, Michelle A. Linterman, Kathryn J. Wood

**Affiliations:** ^1^Transplant Research Immunology Group, Nuffield Department Surgical Sciences, University of Oxford, Oxford, United Kingdom; ^2^Lymphocyte Signalling ISP, Babraham Institute, Cambridge, United Kingdom; ^3^Department of Immunology and Pathology, Monash University, Melbourne, VIC, Australia

**Keywords:** T follicular helper cells, T follicular regulatory cells, immunosuppression, transplantation immunology, germinal centers

## Abstract

**Background:**

T follicular helper (Tfh) cells are key players in the production of antibody-producing B cells *via* the germinal center reaction. Therapeutic strategies targeting Tfh cells are important where antibody formation is implicated in disease, such as transplant rejection and autoimmune diseases. We investigated the impact of the immunosuppressive agent tacrolimus on human Tfh cell differentiation and function in transplant recipients.

**Methods:**

Paired blood and lymph node (LN) samples were obtained from 61 transplant recipients immediately prior to organ implantation. Living-donor recipients received a week of tacrolimus prior to kidney transplantation. Deceased-donor recipients served as controls, as tacrolimus was not administered until after the transplant operation. Flow cytometry was used to compare LN and circulating cell subsets.

**Results:**

The calcineurin inhibitor (CNIs) tacrolimus specifically suppresses both LN Tfh cells and circulating Tfh cells, but not their regulatory counterparts or other CD4 T cell subsets.

**Conclusion:**

Our findings suggest that CNIs may have a more important role in the prevention of antibody formation than previously understood and, therefore, have potential for antibody-associated conditions in which aberrant Tfh function has been implicated in disease.

## Introduction

The germinal center (GC) reaction is required to form long-lasting antibody responses and requires interactions between many different cell types; GC B cells, T follicular helper (Tfh) cells, and T follicular regulatory (Tfr) cells (1, 2). By acting in concert, these cells support the development of memory B cells that can respond quickly to future infections, and long-lived plasma cells that migrate to the bone marrow and secrete high affinity, class-switched antibodies. The long-lived humoral immunity produced from the GC is the cornerstone of successful vaccination, but can result in negative health outcomes when the response is directed to self-antigens, or alloantigens in the context of transplantation (3). Therefore, therapeutic strategies to manipulate the GC response are required in diseases where there is an unmet clinical need to suppress aberrant humoral immunity, for example, in autoimmune disease or transplant rejection.

Investigating the differentiation and function of the cells involved in the GC response and the effect of therapeutic agents is, therefore, important but challenging, due to the scarcity of fresh human secondary lymphoid tissue. Transplant recipients offer a unique opportunity to analyze paired blood and lymph node (LN) samples, enabling direct comparison of circulating and tissue resident populations (4) and the effects of immunosuppressive therapy, as reported here.

This clinical setting is also ideal for determining the effect of immunosuppressive therapy on Tfh cells, and their circulating counterparts. Calcineurin inhibitors (CNIs) are widely used in transplantation, a key mechanism of their action is blockade of NFAT signaling, which is known to be important for Tfh development and differentiation in mice (5). We have taken advantage of the unique opportunity to obtain paired blood and LN samples from the same individual undergoing a transplant to compare the effects of the CNI tacrolimus on both LN and circulating Tfh (cTfh) and Tfr cells. Living-donor kidney transplant recipients at the Oxford Transplant Centre receive 1 week of tacrolimus monotherapy prior to the planned operation. In contrast, deceased-donor transplant recipients receive tacrolimus only after the operation but are otherwise equivalent in having chronic kidney disease stage 5, with its associated generalized immunosuppressive effect (6). We obtained paired blood and LN samples from both groups of patients immediately prior to the operation, allowing comparison of the effects of the CNI tacrolimus on both blood and LN T and B cells.

Because, in humans, access to lymphoid tissue-resident Tfh cells is challenging, most studies have used a circulating biomarker of these cells. Circulating counterparts of Tfh and Tfr cells, described in mice and in humans, share the same surface markers as GC cells and are thought to be memory cells, capable of rapid initiation of antibody responses upon re-encounter with antigen (7–11). Our paired blood and tissue sampling protocol enables direct comparison of LN Tfh and their circulating counterparts. cTfh cells express the chemokine receptor CXCR5, and can be further subdivided into subsets using cytokine receptors that are hallmarks of other helper T cell subsets. cTfh1 cells express CXCR3, and IFNγ. Similarly, cTfh17 cells express CCR6 and secrete IL-17. cTfh2 cells do not express either CXCR3 or CCR6 but can secrete IL-4. These cells have different capacity for B cell help; cTfh2 and cTfh17 are capable of helping both naïve and memory B cells produce antibody *in vitro*; while cTfh1 cells provide help to memory B cells, but not naïve cells. Furthermore, the helper capacity of cTfh1 cells appears to be restricted to those expressing high levels of ICOS and PD-1 (7, 10).

An increased frequency of cTfh cells has been identified in a myriad of human autoimmune diseases; including systemic lupus erythematosus, rheumatoid arthritis, and autoimmune thyroid disease, and appears to correlate with autoantibody level and/or disease severity (12–18). Similar increases in blood CXCR3^+^CXCR5^+^ICOS^+^ Tfh cells occurred 7 days after influenza vaccination and correlated with anti-influenza antibody and plasmablast production (10). An increased proportion of circulating CXCR3^−^CXCR5^+^PD-1^+^ Tfh cells is also seen in those patients with HIV infection who go on to form broadly neutralizing antibodies (9). Taken together, this research indicates that cTfh cells can be a circulating biomarker of GC activity in both health and disease. Because of their role in antibody development and the clinical problem of *de novo* donor-specific antibodies (DSAs) in transplantation, one study identified that patients with pre-formed DSA had increased numbers of circulating CXCR5^+^CD4^+^ cells after transplantation compared to those without, but found no differences in patients developing *de novo* DSA (19). More recently, increased numbers of CXCR5^+^CD4^+^ cells with low PD-1 expression have been described in a small cohort of patients with chronic rejection, compared to those with stable renal function. However the group was heterogeneous with varied immunosuppressive regimens (20). In liver transplantation, no change was seen in the number of CXCR5^+^CD4^+^ cells after transplantation, but their effector function through IL-21 production was reduced (21), consistent with *in vitro* work showing that blockade of IL-21 can prevent alloreactive B cell differentiation (22). Despite the interest in Tfh and Tfr cells in transplantation, little is currently known about how currently used immunosuppressive agents impact the development or function of these cells (23). An *in vitro* model suggested that CNIs may suppress Tfh cell development (24); however, so far, there is little evidence *in vivo*, and it is not clear if these effects are specific to Tfh cells or shared with other T cell subsets.

Here, we report that a week of tacrolimus treatment reduces cTfh cells in the periphery and Tfh precursor cells in the LN without a significant impact on other CD4 T cell subsets. This is supported by *in vitro* work demonstrating that addition of tacrolimus to a Tfh-B cell co-culture prevents B cell maturation and antibody production. These data suggest that tacrolimus could be an effective clinical intervention for targeting Tfh cells in humans.

## Patients and Methods

### Patients

This study was conducted in compliance with Good Clinical Practice and the Declaration of Helsinki, and received ethical approval from the Local Research Ethics Committee, REC reference 14/SC/0091. Written informed consent was obtained from all patients.

Kidney and simultaneous pancreas-kidney (SPK) transplant recipients were recruited over an 8-month period from May to December 2014 from the Oxford Transplant Centre. Patients with known pre-formed donor-specific anti-HLA antibodies and those undergoing planned pre-transplant desensitization with antibody removal were excluded, but those with a negative pre-transplant cross-match to donor HLA were approached for study-specific consent. Details of patients are shown in Table 1. In total, 42 kidney alone and 19 SPK recipients were recruited to the study and provided paired blood and tissue samples. SPK recipients had a median age of 47 (range 30–59) and were evenly sex-matched (9 male, 10 female). Kidney recipients had a median age of 54 (range 26–74) and had a male preponderance (31 male, 11 female). 16 of 42 kidney-alone samples were from live-donor recipients; the remaining 26 kidney-alone and all 19 SPK samples were from deceased donor recipients.

**Table 1:** Table of patient characteristics at recruitment.

	Kidney	Kidney pancreas
Number recruited	42	19
Sex male/female	31M/11F	9M/10F
Median age (range)	54 (26–74)	47 (30–59)

**Donor type**		
Living (male/female)	16 (11M/5F)	n/a
DBD (male/female)	11 (7M/4F)	16 (7M/9F)
DCD (male/female)	15 (13M/ F)	3 (2M/1F)

**Drug treatment regimen**		
Alemtuzumab	19	19
Basiliximab + MMF	13	n/a
Basiliximab + Aza	10	n/a

*61 patients were recruited in total*.

*DBD, donor after brainstem death; DCD, donor after cardiac death; MMF, mycophenolate mofetil; Aza, azathioprine*.

All patients received a dose of an antimetabolite (azathioprine or MMF) 2 h prior to transplantation. Patients receiving kidneys from live-donors also received 1 week of treatment with tacrolimus (aiming for trough levels of 5–15 ng/L) prior to transplantation. Only those patients with no evidence of acute infection at the time of admission went ahead to receive the transplant.

At the time of transplant, 50 ml of blood was taken immediately after induction of anesthesia, and lymphoid tissue was removed prior to organ implantation, to allow access to blood vessels as part of the routine operative procedure. LN from kidney recipients were from the inguinal region; those from SPK recipients were from the para-aortic region.

Data from an independent cohort of patients with end-stage renal failure undergoing kidney transplantation at another transplant center are included in Figure 3. These patients also provided written informed consent to use of their samples for research, and the samples were collected and processed in the same manner as in this study (4).

### Sample Preparation

Blood samples were prepared over a density gradient as previously described and LN samples made into a single cell suspension as previously described (4). Samples were cryopreserved in a mix of 90% fetal calf serum and 10% DMSO, then thawed and stained for flow cytometry in batches to reduce any variation between individual samples that would be introduced from processing. Defrosted samples were washed, counted, and 3 × 10^6^ cells added to each polypropylene tube for flow cytometry.

All samples were incubated with human Fc block (eBioscience) to prevent non-specific antibody binding. A cocktail of surface antibodies was added and samples incubated for 30 min at room temperature. They were washed; streptavidin and live/dead stains were added for a further 10 min at room temperature, samples were then washed again, and fixed using eBioscience Foxp3 staining fixation/permeabilization buffer for 45 min at 4°C as per manufacturers protocol. Antibodies to intracellular proteins were added and samples incubated for 60 min at 4°C. Samples were run on a BD LSR Fortessa cell analyzer. Antibody cocktails were mixed in brilliant violet stain buffer (BD biosciences) to prevent “budding” of the negative population, as recommended by BD bioscience.

### Antibodies

Anti-human CD38 (clone HIT2), CD19 (clone SJ25C1), HLA-DR (clone G46-6), CD45RA (clone HI100), CXCR5 biotin (clone RF8B2), PD-1 (clone EH12.1), IL-7R (clone HIL-7R-M21), Bcl6 (clone K112-91), IgM (clone H1-FB1), CXCR3 (clone IC6), CD57 (clone NK-1), PD-1 (clone EH12.1) CXCR3 (clone 1C6/CXCR3), and CD27 (clone M-T271), all BD Biosciences. Anti-human IgD (clone IA6-2), CCR7 (clone 3D12), CCR6 (clone R6H1), CD27 (clone O323) and CD4 (clone OKT4), all eBioscience. Anti-human CD24 (clone ML5), ICOS (clone C398.4A), Foxp3 (clone 259D), CD25 (clone M-A251), streptavidin brilliant violet 605, CD4 (clone OKT4), CXCR5 (clone J252D4), CD3 (clone OKT3), CD19 (clone HIB19), and DAPI, all BioLegend. Invitrogen near-IR fixable live-dead stain kit.

### Tfh and B-Cell Co-Cultures

Cells for *in vitro* co-cultures were obtained from leukocyte cones from platelet donors attending the Oxford Blood Donation Centre at the John Radcliffe hospital. Original consent to use of samples was provided to NHS Blood and Transplant, and samples made available for research under local HTA licensing. Isolated PBMCs were pre-enriched for B cells (CD19 Dynabead/Detatchabead kit, Invitrogen) and CD4^+^ T cells (Miltenyi CD4 negative isolation kit). CD19^+^CD27^+^ memory B cells and CXCR5^+^CXCR3^+^ Tfh1, CXCR5^+^CXCR3^−^ Tfh2/17 or CXCR5^−^CXCR3^+/−^ Teffector cells were flow-sorted from pre-enriched populations and then cultured in 96-well *U*-bottom tissue culture plates (3 × 10^4^ of each cell type per well) in the presence of 1 µg/ml staphylococcal enterotoxin B (SEB, Sigma). After 11 days of co-culture, supernatants were retrieved for ELISA and replicate wells combined for flow cytometry of cells.

For drug cultures, tacrolimus (Sigma F4679) solubilized in DMSO was used at 8 ng/ml. This is the mid-range of recommended dosing for patients (5–15 ng/ml) (25, 26) and in keeping with previous *in vitro* work (27). Control samples were run with DMSO vehicle alone.

### IgG and IgM ELISA

Secretion of IgM and IgG was determined by ELISA using the eBioscience Ready-Set-Go! ELISA kits for human total IgG (eBioscience) and human IgM (eBioscience), using polystyrene high-binding 96-well plates, according to eBioscience recommended protocol.

### Statistical Analysis

Statistical analysis was performed using Graph Pad Prism software. Patient samples were analyzed with the Mann–Whitney test. Co-culture samples were analyzed with one-way ANOVA comparing treated to untreated samples with either Bonferroni or Dunnett’s multiple comparison test. Absolute cell counts for peripheral blood samples were calculated using the proportion of lymphocytes for each subset multiplied by hospital laboratory lymphocyte counts taken at the same time as the original sample.

## Results

### Pre-Transplant Tacrolimus Treatment Significantly Reduces cTfh Cell Number

Patients were recruited into the study at the time of transplant; and were, therefore, all either receiving or within 6 months of requiring renal replacement therapy. Those who had received a transplant from a living-donor (*n* = 16) had been treated with the CNI tacrolimus for 1 week prior to transplantation, aiming for trough levels of 5–10 ng/ml. Samples were compared with patients receiving a deceased-donor kidney or SPK transplant (total *n* = 45) who had not received any CNI at the time of sample acquisition. Details are shown in Table 1.

**Figure 1:**
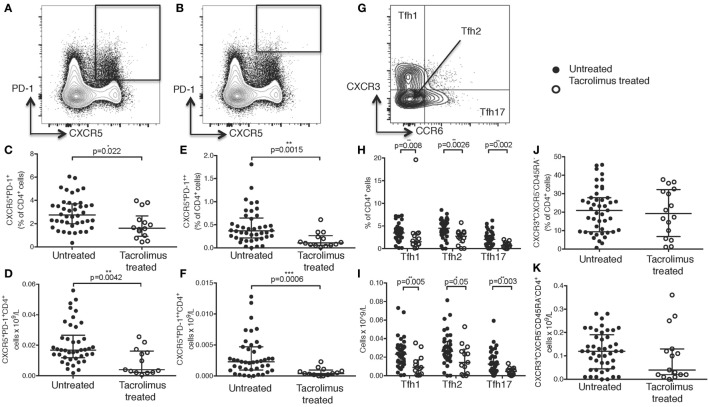
Tacrolimus treatment reduces circulating Tfh frequency. Samples were gated on lymphocytes then live CD4^+^ cells. **(A)** T follicular helper (Tfh) were identified as CXCR5^+^PD-1^+^CD4^+^, with activated Tfh **(B)** identified as CXCR5^+^PD-1^++^CD4^+^. CXCR5^+^PD-1^+^CD4^+^ Tfh shown as proportion **(C)** and absolute count **(D)** in live donor recipients who had received a week of tacrolimus (tacrolimus-treated, *n* = 16) compared to untreated deceased donor recipients (untreated, *n* = 45). CXCR5^+^PD-1^++^CD4^+^ activated Tfh shown as proportion **(E)** and absolute count **(F)**. **(G)** CD4^+^CXCR5^+^ cells were subdivided into CXCR3^+^CCR6^−^ Tfh1, CXCR3^−^CCR6^−^ Tfh2 and CXCR3^−^CCR6^+^ Tfh17 and are shown as both proportion of CD4^+^cells **(H)** and absolute cell count **(I)** in tacrolimus-treated patients compared to untreated. There were no reductions in any other CD4 T cell subset, CXCR3^+^CXCR5^−^CD45RA^−^ Th1 cells are shown as an example with **(J)** proportion of CD4^+^ and **(K)** absolute cell count. Shown as median and interquartile range, each symbol represents one patient. Samples compared with Mann–Whitney test, only significant differences are shown on graphs.

CD45RA is a surface marker to distinguish naïve (CD45RA^+^) from activated/memory (CD45RA^−^) cells, which include cTfh cells. Lack of CD45RA is, therefore, often used in gating cTfh cells. However, a genetic variant exists, where CD45RO is expressed alongside CD45RA, instead of the latter being downregulated (28–30), meaning CD45RA cannot be used to distinguish naïve from memory cells in those with the genetic variant. Three patients in this study had this mutation; therefore, an alternative gating strategy for cTfh cells was sought (Figure 1A).

In this cohort of patients, peripheral blood CXCR5^+^PD-1^+^CD4^+^ cTfh cells (Figure 1A) were significantly reduced in both proportion (Figure 1C, *p* = 0.022) and absolute cell count (Figure 1D, *p* = 0.0042) in tacrolimus treated patients compared to untreated patients. Activated CXCR5^+^PD-1^++^CD4^+^ Tfh cells (31) (Figure 1B) were also significantly reduced in tacrolimus treated patients compared to untreated patients both in proportion (Figure 1E, *p* = 0.0015) and absolute number (Figure 1F, *p* = 0.0006).

In line with previous studies (7), CXCR5^+^CD4^+^ T cells were divided into CXCR3^+^CCR6^−^CXCR5^+^CD4^+^ Tfh1, CXCR3^−^CCR6^−^CXCR5^+^CD4^+^ Tfh2, and CXCR3^−^CCR6^+^CXCR5^+^CD4^+^ Tfh17 subsets (Figure 1G). There was a global reduction in cTfh cells, with all subsets being reduced in tacrolimus treated patients, both in proportion (Figure 1H) and absolute count (Figure 1I), suggesting that tacrolimus does not act selectively on cTfh cell subsets. Importantly, the effect of tacrolimus was specific to Tfh cells; there was with no difference in either proportion (Figure 1J) or absolute count (Figure 1K) of CXCR3^+^CXCR5^−^CD45RA^−^CD4^+^ Th1 cells in tacrolimus treated compared to untreated patients. These results indicate a selective effect of tacrolimus on cTfh cells compared to other T cell subsets.

### Pre-Transplant Tacrolimus Does Not Impact on Peripheral or LN B Cell Subsets

**Figure 2:**
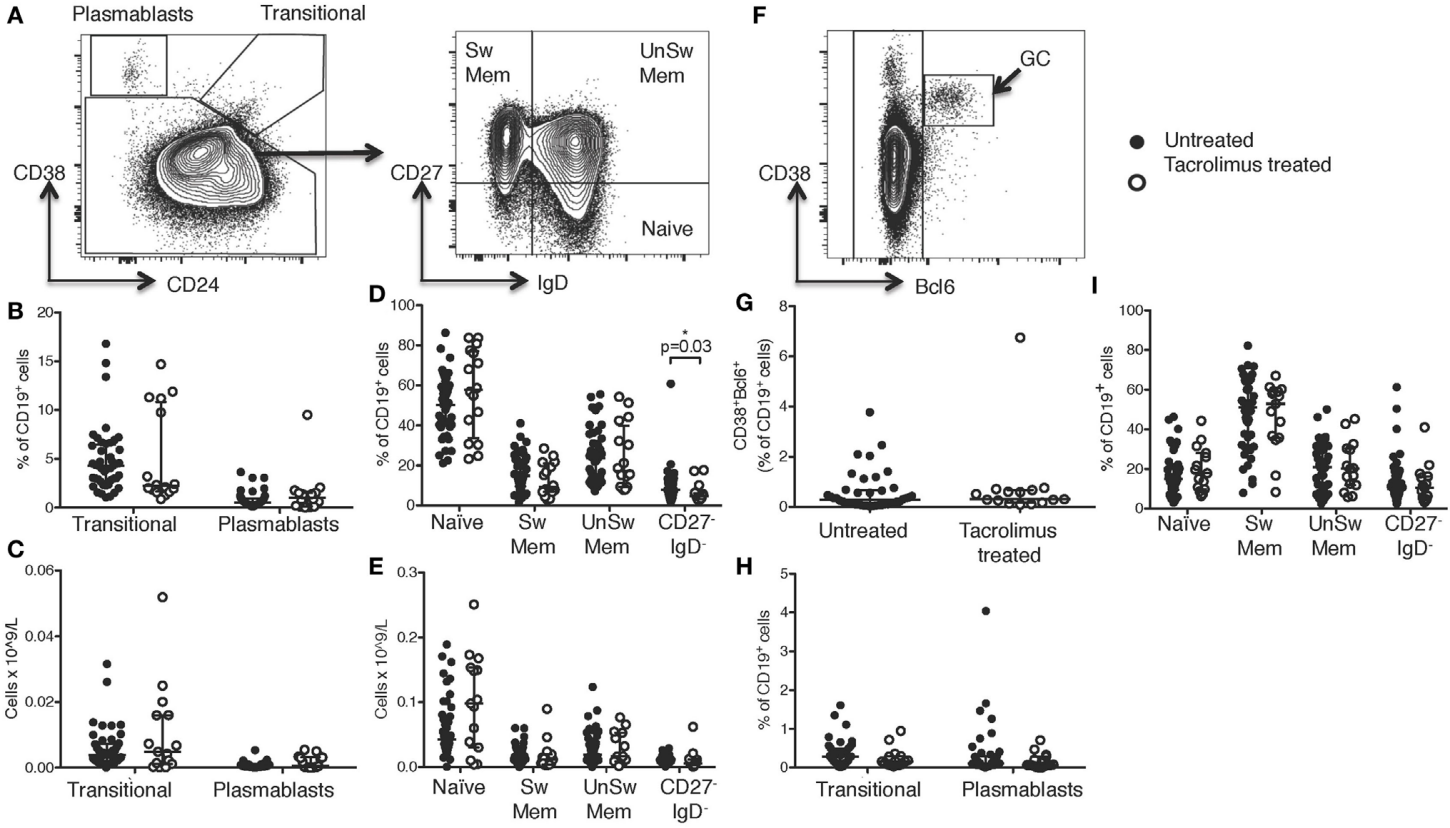
Tacrolimus treatment does not impact B cell numbers. **(A)** Gating strategy for peripheral blood B cells. CD38^hi^CD24^−^ plasmablasts and CD38^hi^CD24^hi^ transitional cells shown as proportion of B cells **(B)** and absolute cell count **(C)** comparing deceased donor recipients, receiving no immunosuppression (untreated, *n* = 45) and live donor recipients receiving a week of tacrolimus (tacrolimus-treated, *n* = 16). CD27^+^IgD^−^ class-switched memory cells, CD27^+^IgD^+^ unswitched memory cells, CD27^−^IgD^+^ naïve cells and CD27^−^IgD^−^ cells shown as proportion of B cells **(D)** and absolute cell count **(E)** comparing untreated to tacrolimus-treated patients. **(F)** For lymph node B cells, CD38^hi^Bcl6^+^ germinal center (GC) cells were gated first, then CD38^hi^CD24^−^ plasmablasts, CD38^hi^CD24^hi^ transitional cells, CD27^+^IgD^−^ switched memory cells, CD27^+^IgD^+^ unswitched memory cells, CD27^−^IgD^+^ naïve cells and CD27^−^IgD^−^ double negative cells as per peripheral blood B cells. **(G)** CD38^hi^Bcl6^+^ GC B cells in deceased donor recipients, receiving no immunosuppression (untreated, *n* = 45) and live donor recipients receiving a week of tacrolimus (tacrolimus-treated, *n* = 16). **(H)** CD38^hi^CD24^hi^ transitional cells and CD38^hi^CD24^−^ plasmablasts in untreated and tacrolimus-treated patients. **(I)** CD27^+^IgD^−^ class-switched memory cells, CD27^+^IgD^+^ unswitched memory cells, CD27^−^IgD^+^ naïve cells and CD27^−^IgD^−^ cells in untreated and tacrolimus-treated patient. Shown as median and interquartile range, each symbol represents one patient. Analyzed with Mann–Whitney testing, only significant differences are shown on graphs.

Because cTfh cells are known to correlate with the frequency of circulating plasma cells in the context of vaccination (10), we wished to determine if there were any alterations in the circulating B cell subsets after CNI treatment. Peripheral blood samples were gated as shown in Figure 2A, allowing comparison of CD38^hi^CD24^−^CD19^+^ plasmablasts, CD38^hi^CD24^hi^CD19^+^ transitional cells, CD27^+^IgD^−^CD19^+^ class-switched memory cells, CD27^+^IgD^+^CD19^+^ unswitched memory cells, CD27^−^IgD^+^CD19^+^ naïve cells, and CD27^−^IgD^−^CD19^+^ cells.

Comparing tacrolimus treated with untreated patients, there was no significant difference in CD38^hi^CD24^−^CD19^+^ plasmablasts or CD38^hi^CD24^hi^CD19^+^ transitional cells either in proportion of CD19^+^ cells (Figure 2B) or absolute cell count (Figure 2C). There was a slight reduction in the proportion of CD19^+^ cells falling into the CD27^−^IgD^−^ gate (*p* = 0.03, Figure 2D), but no difference in absolute cell count (Figure 2E) and no differences in CD27^+^IgD^−^CD19^+^ class-switched memory cells, CD27^+^IgD^+^CD19^+^ unswitched memory cells, or CD27^−^IgD^+^CD19^+^ naïve cells.

We then sought to determine whether there were alterations in the LN-resident B cell populations. In LN, B cell subsets were identified as CD38^hi^BCL6^+^CD19^+^ GC B cells, non-GC B cells were then divided into the same subsets as peripheral blood B cells, consistent with the analyses shown in Figure 2A (gating strategy for LN cells shown in Figure 2F). Comparison of LN B cells from tacrolimus treated and untreated patients showed no significant difference in any B cell subset (Figures 2G–I). These data demonstrate that tacrolimus does not alter the frequency of B cell subsets in the LN. Of note, there were very few LNs with a GC reaction (Figure 2G), which likely reflects the selection of a patient cohort without an ongoing immune response in LN. This is consistent with our previous work (4), likely because infection is a contraindication to proceed with transplantation.

Together, these data show that there were no significant differences in absolute B cell numbers in the circulation or the proportion of LN B cells between live-donor recipients treated with tacrolimus for 1 week (tacrolimus treated patients) compared to deceased donor recipients with no tacrolimus treatment (untreated patients).

### Pre-Transplant Tacrolimus Reduces LN CXCR5^+^PD-1^+^ Tfh Cells

As cTfh cells are considered to be a biomarker of LN Tfh activity (8–10, 12–18), we were interested in whether the reductions seen in cTfh cells in our patient cohort corresponded to alterations in LN Tfh cells. Within the LN, CXCR5^+^CD4^+^ T cells are a diverse group of cells, encompassing Tfh cell precursors, which have not yet entered the GC; fully differentiated GC-located Tfh; and antigen-specific memory Tfh, which express lower levels of co-stimulatory markers such as ICOS and PD-1 but are capable of rapid recall responses on antigen restimulation (32). To determine whether tacrolimus treatment had an impact on LN Tfh cells similar to cTfh cells, we analyzed multiple subsets of Tfh cells within the LN.

**Figure 3:**
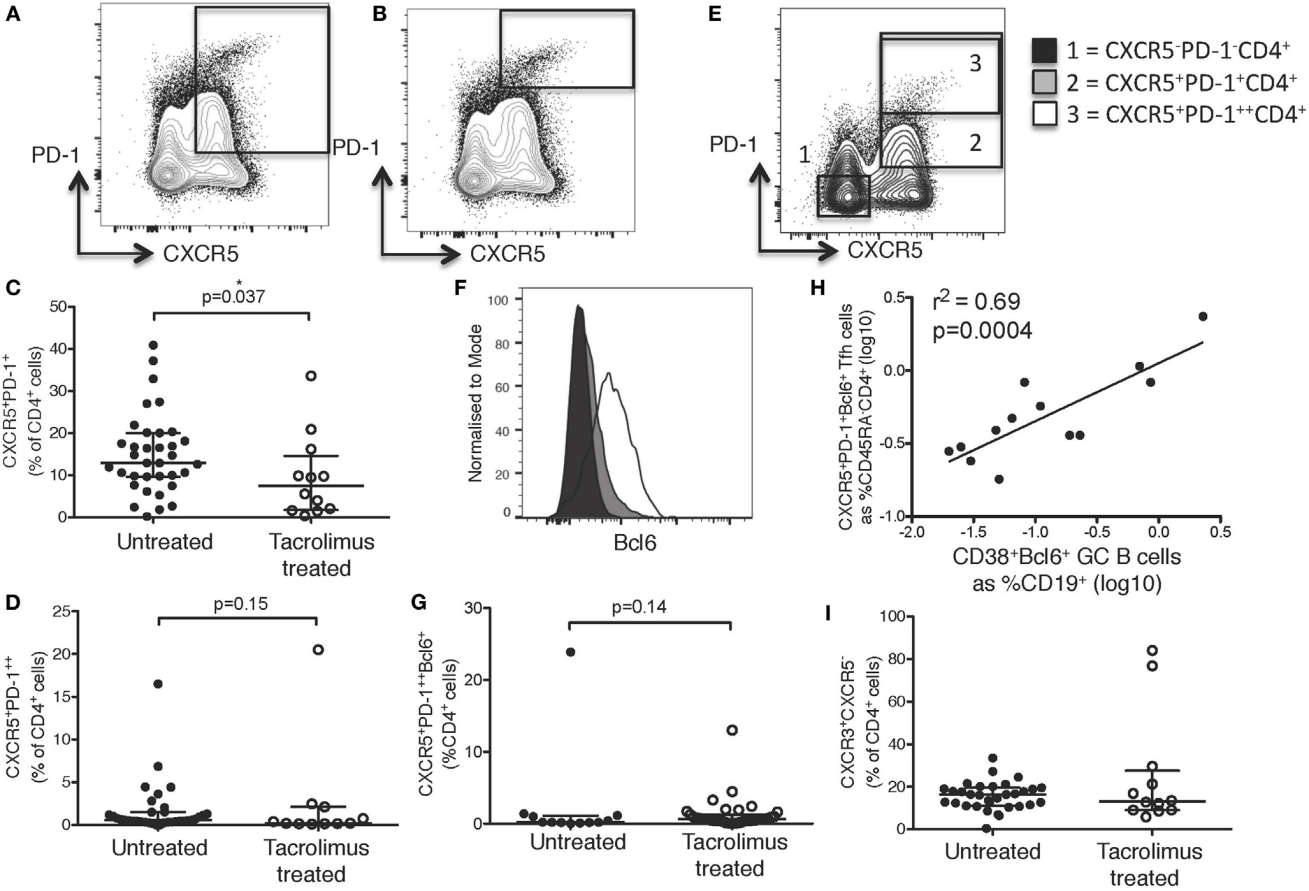
Tacrolimus reduces lymph node (LN) T follicular helper (Tfh) cells. Samples were gated on lymphocytes then live CD4^+^ cells. LN Tfh were identified as **(A)** CXCR5^+^PD-1^+^ with germinal center (GC) Tfh separated out as **(B)** CXCR5^+^PD-1^++^. **(C)** LN CXCR5^+^PD-1^+^ Tfh shown in live donor recipients who had received a week of tacrolimus (tacrolimus treated, *n* = 16) compared to untreated deceased donor recipients (untreated, *n* = 45). **(D)** GC CXCR5^+^PD-1^++^ Tfh shown in tacrolimus treated and untreated. **(E)** Gating for Bcl6 expression on CD4^+^ cells. **(F)** Fluorescence intensity of Bcl6 on CXCR5^−^PD-1^−^CD4^+^ non-Tfh cells, CXCR5^−^PD-1^+^CD4^+^ Tfh and CXCR5^−^PD-1^++^CD4^+^ GC located Tfh cells. **(G)** CXCR5^+^PD-1^++^Bcl6^+^ Tfh in untreated compared to tacrolimus treated patients. **(H)** Pearson correlation between CXCR5^+^PD-1^+^Bcl6^+^CD45RA^−^CD4^+^ Tfh and CD38^+^PD-1^+^Bcl6^+^CD19^+^ GC B cells. **(I)** CXCR3^+^CXCR5^−^CD4^+^ Th1 cells in LNs of untreated and tacrolimus treated patients. Shown as median and interquartile range, each symbol represents one patient. Analysed with Mann Whitney testing, only significant differences are shown on graphs.

Broadly, Tfh cells were identified in the LN as CXCR5^+^PD-1^+^CD4^+^ cells (Figure 3A) with GC located Tfh identified as the CXCR5^+^PD-1^++^CD4^+^ subset (Figure 3B). CXCR5^+^PD-1^+^CD4^+^ cells showed a significant reduction (*p* = 0.037) in tacrolimus treated compared to untreated patients (Figure 3C). However, CXCR5^+^PD-1^++^CD4^+^ GC Tfh were not significantly different (*p* = 0.15) between tacrolimus treated and untreated patients (Figure 3D). Bcl6 expression was highest in CXCR5^+^PD-1^++^CD4^+^ GC Tfh cells, and intermediate in CXCR5^+^PD-1^+^CD4^+^ Tfh cells (Figures 3E,F), CXCR5^+^PD-1^++^Bcl6^+^CD4^+^ GC Tfh were not significantly different (*p* = 0.14) between tacrolimus treated and untreated patients (Figure 3G). This likely reflects the quiescent nature of the LNs in this patient cohort, as there were very few GCs in the LNs of these patients (Figure 2G). In support of this, data from an independent cohort of inguinal LN samples from our laboratory show that there is a significant correlation (*r*^2^ = 0.69, *p* = 0.0004) between the number of CXCR5^+^PD-1^++^Bcl6^+^CD4^+^ GC Tfh cells and the number of GC B cells (Figure 3H) in human LN. It may also reflect the relatively short duration of tacrolimus therapy at the point of analysis, 1 week, suggesting that the majority of effects are on Tfh precursors, or memory Tfh with low level PD-1 expression, rather than GC-localized Tfh cells. Together, these data suggest that tacrolimus can inhibit human Tfh cell differentiation.

As with cTfh cells, LN CXCR5^+^CD4^+^ T cells were divided into CXCR3^+^CCR6^−^CXCR5^+^CD4^+^ Tfh1, CXCR3^−^CCR6^−^CXCR5^+^CD4^+^ Tfh2, and CXCR3^−^CCR6^+^CXCR5^+^CD4^+^ Tfh17 subsets, but there were no differences in the proportion of any subset of Tfh cells (data not shown). As seen in peripheral blood, there were no significant differences in any CXCR5^−^CD4^+^ cell subsets, including CXCR3^+^CXCR5^−^CD4^+^ Th1 cells, shown as an example (Figure 3I).

While GC located Tfh expressing the highest levels of PD-1 were not diminished by tacrolimus treatment, given the overall reduction in both LN Tfh cell precursors and cTfh cells, these data support previous work that cTfh cells are a biomarker of LN Tfh cells (4, 9, 10, 33, 34) and is important for the clinical follow-up of these patients, as only blood samples are available after transplantation. Interestingly, unlike GC Tfh, the reduction in CXCR5^+^PD-1^++^CD4^+^ in the peripheral blood may suggest that cTfh-like cells that resemble GC-Tfh cells are more sensitive to calcineurin blockade than GC Tfh. However, this may be due to the fact LN that are sampled during surgery contain very few GCs, and the cTfh cells reflect the cumulative responses of all secondary lymphoid organ GC responses, particularly those in the gut that have persistent GC reactions, which were not sampled in these patients.

### Pre-Transplant Tacrolimus Does Not Impact on Treg or Tfr Cells

**Figure 4:**
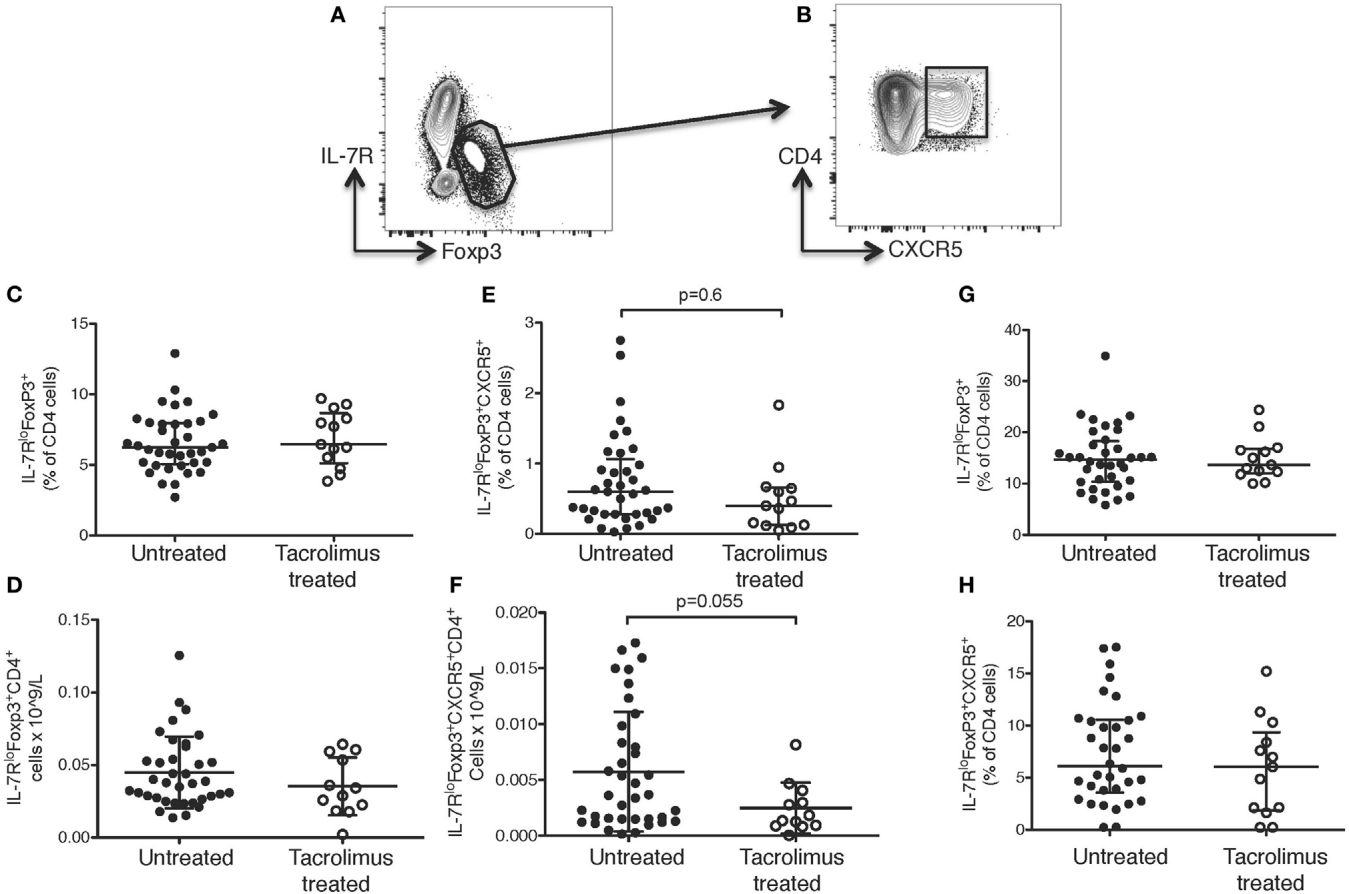
Tacrolimus does not impact T follicular regulatory (Tfr) or regulatory T cells. Samples were gated on lymphocytes then live CD4^+^ cells. **(A)** Regulatory T cells (Tregs) were gated as IL-7R^lo^Foxp3^+^, then **(B)** Tfr cells were gated as CXCR5^+^ Tregs. There were no significant differences between Tregs or Tfr in untreated deceased donor recipients (untreated) and live donor recipients (tacrolimus treated) in blood either in proportion of cells **(C)** or absolute cell count **(D)**. There was a trend toward lower Tfr in peripheral blood both in proportion of cells **(E)** and absolute cell count **(F)**, but this did not reach statistical significance on Mann–Whitney testing. There were no significant differences in lymph node Tregs **(G)** or Tfr cells **(H)** between untreated and tacrolimus treated patients. Shown as median and interquartile range, each symbol represents one patient, *n* = 45 untreated, *n* = 16 tacrolimus treated. All samples analyzed with Mann–Whitney testing.

Given the differences seen in Tfh cells, we sought to determine if their regulatory counterparts were altered with tacrolimus treatment, as regulatory T cells (Tregs) can be reduced by CNIs (35–38). Tregs were identified as IL-7R^lo^FOXP3^+^CD4^+^ cells using the gating strategy shown in Figure 4A. Tfr cells should express both Treg and Tfh surface markers and were, therefore, identified as CXCR5^+^IL-7R^lo^FOXP3^+^CD4^+^ cells (Figure 4B).

There were no significant differences in CD4^+^ T cells as a whole (data not shown) or in IL-7R^lo^FOXP3^+^CD4^+^ Tregs in the peripheral blood in either proportion of cells (Figure 4C) or absolute count (Figure 4D) in tacrolimus treated patients compared to untreated patients. There was a trend toward fewer CXCR5^+^IL-7R^lo^FOXP3^+^CD4^+^ Tfr cells in the peripheral blood of tacrolimus treated patients both in proportion of CD4^+^ cells (Figure 4E) and absolute cell count (Figure 4F), but this did not reach statistical significance. There were no significant differences in LN IL-7R^lo^FOXP3^+^CD4^+^ Tregs (Figure 4G) or LN CXCR5^+^IL-7R^lo^FOXP3^+^CD4^+^ Tfr cells (Figure 4H) between tacrolimus treated and untreated patients. These data suggest that suppression of Tfr cells, like Tregs, may require higher doses or longer duration of CNI treatment than required for suppression of Tfh cells (35–38).

### Tacrolimus Treatment Reduces Tfh Cell Activation, Plasmablast Differentiation, and Antibody Production *In Vitro*

**Figure 5:**
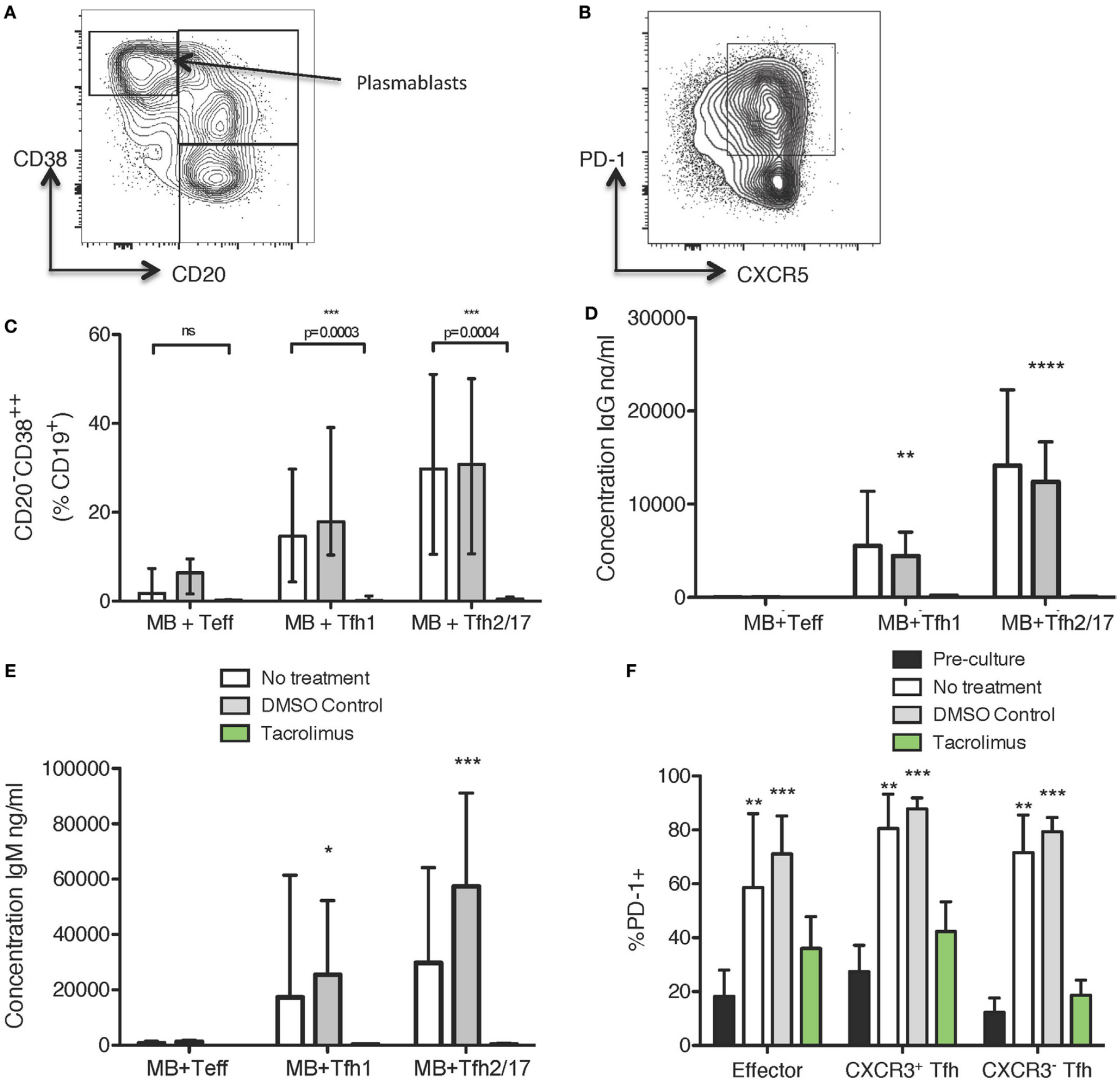
Tacrolimus reduces plasmablast formation and antibody production in B cell/Tfh co-cultures. **(A)** Gating strategy for CD19^+^ cells. **(B)** Gating strategy for CXCR3^+/−^CD4^+^ cells. **(C)** CD20^−^CD38^++^ differentiation in CD27^+^ memory B cells cultured with CXCR5^−^CD4^+^ Teffectors, CXCR3^+^CXCR5^+^CD4^+^ Tfh1, or CXCR3^−^CXCR5^+^CD4^+^ Tfh2/17, showing tacrolimus treated co-cultures compared to untreated or DMSO control. IgG **(D)** and IgM **(E)** production in tacrolimus treated co-cultures compared to untreated or DMSO control. **(F)** Upregulation of PD-1 on CXCR5^−^CD4^+^ Teffectors, CXCR3^+^CXCR5^+^CD4^+^ Tfh1, or CXCR3^−^CXCR5^+^CD4^+^ Tfh2/17 in untreated, DMSO control, or tacrolimus treated co-cultures, compared to pre-culture expression. For all samples, the height of the bar represents the mean of two technical replicates from four biological replicates, the error bar shows SD. All samples analyzed with one-way ANOVA comparing treated to untreated followed by Bonferroni multiple comparison test, except *F* where all post-culture conditions were compared to pre-culture PD-1 expression using Dunnett’s multiple comparison test. *p < 0.05, **p < 0.01, ***p < 0.001, ****p < 0.0001.

To determine if tacrolimus acts directly on Tfh cell activation and helper capacity, we used a previously described (7, 34) *in vitro* system to test Tfh cell function, which allows assessment of B helper capacity. Peripheral blood CD19^+^CD27^+^ memory B cells were co-cultured for 11 days with CXCR5^+^CXCR3^+^CD4^+^ Tfh1, CXCR5^+^CXCR3^−^CD4^+^ Tfh2/17 or, as a negative control, CXCR5^−^CXCR3^+/−^CD4^+^ Teffectors. The culture medium contained no drug, vehicle control (DMSO), or tacrolimus; therefore, all therapy was present throughout the 11-day culture. Flow cytometry at the end of culture allowed assessment of B cell alterations with expression of CD38 and loss of CD20 used as a marker of plasmablast differentiation (Figure 5A) and of CD4 T cell alterations with expression of PD-1 as an activation marker (Figure 5B). Co-culture of CD19^+^CD27^+^ memory B cells with Tfh1 and Tfh2/17 led to CD20^−^CD38^hi^CD19^+^ plasmablast differentiation in untreated and vehicle treated control co-cultures (Figure 5C), while tacrolimus treatment significantly impaired plasmablast formation. This lack of plasmablast differentiation was reflected in the absence of IgG (Figure 5D) and IgM (Figure 5E) production in the supernatant of tacrolimus treated co-cultures compared to untreated and vehicle control. We could not perform this analysis on B cells alone, as B cells cultured with SEB in the absence of T cells did not survive in culture irrespective of drug treatment.

The phenotype of cTfh cells altered following co-culture. In untreated and vehicle-control treated co-cultures, PD-1 expression was significantly upregulated (Figure 5F), consistent with the cTfh cells becoming activated during co-culture. However, this activation was abrogated by tacrolimus treatment, with PD-1 expression on Teffectors, Tfh1, and Tfh2/17 not significantly different compared to pre-culture levels (Figure 5F). This suggests a block in T cell activation, which would limit interaction with B cells, thus preventing antibody production. Together, these data suggest that tacrolimus in co-culture conditions blocks the activation and proliferation of Tfh cells, which limits the provision of help to B cells, and subsequent antibody production; however, we cannot exclude a direct effect on B cells.

## Discussion

T follicular helper cells are essential for long-lived humoral immunity, making them critical mediators of both health and disease. They are essential for protective immunity following vaccination, but have detrimental effects where the production of antibodies is undesirable, such as autoimmune disease. For this reason, treatments that can specifically suppress Tfh cell differentiation and/or function are needed to treat humoral auto- and allo-immunity. Here, we describe that the CNI tacrolimus specifically suppresses human Tfh cell differentiation *in vivo* and alters B cell helper function *in vitro*. These data suggest that tacrolimus may be a therapeutic option for those conditions where clinically undesirable antibody responses are generated. Unfortunately, due to the confounding effects of induction immunosuppression and treatment of all patients posttransplant with tacrolimus as well as additional maintenance agents, it is not possible to say whether these early reductions in Tfh cells would have an impact on the risk of *de novo* DSA formation posttransplant in this patient cohort.

Calcineurin inhibitors have been used in transplantation since their discovery in the 1970s. Their mechanism has been well described (39), as calcineurin is a key phosphatase in TCR signaling. When the TCR engages with peptide–MHC complex, an intracellular calcium flux leads to the activation of calcineurin, leading to the de-phosphorylation of NFAT, which then translocates to the nucleus and promotes transcription of pro-inflammatory cytokines and costimulatory molecules. By binding to immunophilins, CNIs form complexes blocking the activation of calcineurin and hence downstream NFAT-dependent transcription. While B cells can also signal through calcineurin, they are less dependent on this pathway to mediate downstream effects of BCR engagement than T cells (40, 41). The key effect of this blockade is the reduction in IL-2, a cytokine that increases T cell activation and proliferation, recent animal models have suggested that Tfh cells are more dependent on NFAT signaling than other CD4 T cell subsets (5). This is consistent with our observation that cTfh cells, but not peripheral blood Th1 cells, are reduced after tacrolimus treatment. This is likely because NFAT is expressed at higher levels in Tfh cells than in other CD4 subsets (42) and calcium flux in response to TCR engagement has been shown in animal models to be higher in Tfh than Th1 cells (43) with blockade of NFAT signaling *via* ciclosporin abrogating IL-21 production. Martinez et al. showed that in NFAT1- and NFAT2-deficient mice, both Tfh and GC B cells were reduced and antibody responses to viral infection were impaired (5). This was associated with reduced expression of ICOS, PD-1, CD40L, and SLAM, key molecules associated with Tfh differentiation and function (1). The differentiation of other CD4 T cell subsets was not impaired by CD4-specific loss of NFAT, supporting the data we have seen in transplant recipients. These data suggest that Tfh cells are more dependent on NFAT signaling than other CD4 subsets, both for activation *via* ICOS and CD40L interaction, as well as downstream effector mechanisms *via* IL-21 production.

In support of a similar role for NFAT in humans, almost half of genes differentially upregulated in Tfh cells are associated with NFAT1-binding sites (5). Our data support these findings, with calcineurin blockade by tacrolimus leading to a selective reduction in Tfh cells when compared to Tregs and Th1 cells. However, we did not observe defects in GC B cell frequency in our patients, likely because the LN assessed in our study contained very few GC B cells, indicative of lack of antigen stimulation. This is not surprising as active infection at the time of admission is a contraindication to transplantation. Correspondingly, we observed very low numbers of CXCR5^+^PD-1^++^ GC Tfh in the LN, reflecting the non-reactive nature of these LNs in patients without active GC reactions.

Despite the low numbers of GC Tfh cells, we saw a significant reduction in the frequency of LN Tfh cells in tacrolimus-treated patients. These data support the observation from animal models that Tfh cells are more dependent on NFAT signaling, and hence, more sensitive to CNIs, than other CD4 T cell subsets. This has implications for transplantation, as effective calcineurin blockade may have a greater impact on formation of *de novo* DSA than previously thought. Previous studies have shown a global reduction in Tfh with tacrolimus treatment (44), although this was not significantly different to the reduction seen with other immunosuppressive agents, in particular, belatacept, a CTLA-4 Ig fusion protein that blocks costimulation and hence limits T cell activation (44, 45). Interestingly, de Graav et al. also found that tacrolimus blocked *in vitro* plasmablast differentiation, in keeping with our data (44).

It is well known from epidemiological studies that inadequate levels of CNI, whether from non-concordance (46–49) or underdosing (50, 51), are associated with increased risk of *de novo* DSA formation, especially after graft failure, where withdrawal of immunosuppression is strongly associated with DSA formation (52, 53). This emphasizes the need for biomarkers to identify those patients most at risk of *de novo* DSA formation and allow selective adjustment of immunosuppression. Our data provide a possible explanation for the importance of CNIs and supports the suggestion (23) that cTfh cells could be used as a more selective biomarker to differentiate which patients are adequately immunosuppressed and, therefore, at lower risk of developing *de novo* DSA, rather than using trough drug levels, which may over- or under-estimate the immunosuppression of an individual patient.

In summary, the CNI tacrolimus appears have a greater impact on Tfh cells than other CD4^+^ T cell subsets making it of therapeutic interest in both transplantation and autoimmune disease, where increased cTfh cell frequency and an active GC reaction have been associated with autoantibody levels and end organ damage.

## Ethics Statement

This study was conducted in compliance with Good Clinical Practice and the Declaration of Helsinki, and received ethical approval from the Local Research Ethics Committee, REC reference 14/SC/0091. Written informed consent was obtained from all patients.

## Author Contributions

EW designed and performed research, analyzed data, and wrote the paper. DH performed research, analyzed data, and reviewed the paper. ML and KW designed research and wrote the paper.

## Conflict of Interest Statement

The authors declare that the research was conducted in the absence of any commercial or financial relationships that could be construed as a potential conflict of interest.
